# Impact of financial burden, resulting from prescription co-payments, on antihypertensive medication adherence in an older publically insured population

**DOI:** 10.1186/s12889-018-6209-8

**Published:** 2018-11-20

**Authors:** Paul Dillon, Susan M. Smith, Paul Gallagher, Gráinne Cousins

**Affiliations:** 1 School of Pharmacy, RCSI, St. Stephen’s Green, Dublin 2, Ireland; 2 Primary Care Medicine, Department of General Practice and HRB Centre for Primary Care Research, RCSI, St. Stephen’s Green, Dublin 2, Ireland

**Keywords:** Antihypertensive medication, Adherence, Compliance, Co-payments, Medication cost-sharing, Health policy, Financial burden, Older adults

## Abstract

**Introduction:**

Medication co-payments represent a financial barrier to antihypertensive medication adherence. The introduction of co-payments for Irish publically insured patients was associated with a 5% reduction in adherence. However there is socioeconomic variability within this population, and the impact may be greater for those on lower income. We evaluated medication-related financial burden of the co-payment in a cohort of Irish publically insured antihypertensive users and tested its association with adherence at 12 months.

**Methods:**

This was a prospective cohort study of community dwelling older (> 65 yrs) adults (*n* = 1152) from 106 Irish community pharmacies. Participants completed a structured telephone interview at baseline, and a follow-up interview at 12-months, which we linked to pharmacy records. We assessed medication-related financial burden at baseline using a single questionnaire item, and adherence at 12 months via questionnaire and refill-adherence as Proportion of Days Covered (PDC).

**Results:**

A third of participants (30.1%) reported financial burden due to medication costs. In adjusted linear regression models financially burdened participants had significantly lower self-reported adherence (*β* = − 0.29, 95% CI -0.48 to − 0.11), although this was not evident with PDC (*β* = − 2.76, 95% CI -5.65 to 0.14).

**Conclusion:**

This co-payment represents a financial barrier to antihypertensive adherence for many older Irish publically insured patients. The negative impact to adherence will potentially increase the risk of adverse outcomes, such as stroke, and increase long-term healthcare costs.

**Electronic supplementary material:**

The online version of this article (10.1186/s12889-018-6209-8) contains supplementary material, which is available to authorized users.

## Background

Medication co-payments are a cost-sharing policy that intend to reduce the costs of third-party payers by dis-incentivising the use of unnecessary medication and shifting some of the cost-burden to patients [1, 2]. However, co-payments represent a financial barrier to adherence to essential medication [3, 4], and may be disadvantageous if they lead to a decrease in the use of cost-effective medication [5]. The most recent Cochrane review of co-payment policies concluded that reductions in essential medication use may be associated with these policies [2]. A systematic review, which focused solely on publically insured patients, quantified an 11% increased risk of non-adherence associated with co-payments. These co-payment polices ranged from $2 per item up to the full cost of the medication [6]. In 2010, in an attempt to reduce the overall healthcare budget in the face of increasing pharmaceutical expenditure and economic crises, the Republic of Ireland introduced co-payments for medication dispensed to publically insured patients under the General Medical Services scheme (GMS) (see Table 1) [7].

**Table 1:** Eligibility criteria and co-payment levels for the General Medical Services (GMS) scheme

Weekly income thresholds by age group since Jan 1st 2014		Co-payment levied per each prescription item dispensed, maximum monthly ceiling and associated decreases in antihypertensive adherence			
Age (Years)	Weekly Income (€)	Date of introduction/increase	Co-payment (€)	Ceiling (€)	Decrease in Adherence (%)
< 66	266.50	October 2010	0.50	10.00	4.8
66–69	298.00	January 2013	1.50	19.50	4.5
70+	900.00	December 2013	2.50	25.00	–

In the Republic of Ireland, publically insured patients are covered under the GMS scheme. Eligibility for the GMS is means-tested based on income, with approximately 35% of the population covered. Coverage rises to approximately 50% for the population aged between 65 and 69 years and increases to 90% for the population 70 years and over (Central Statistics Office, 2013). Weekly income eligibility thresholds are based on a couple (married/cohabiting/civil partners). Non-GMS patients include Long-term Illness scheme patients (which covers conditions such as diabetes and epilepsy, but not hypertension) who are entitled to free medication, Doctor Visit Card holders (eligibility based on higher weekly thresholds) and private patients who pay for dispensed medications up to maximum of €144 per month. GMS patients are entitled to free primary-care medical visits but pay a levy for each dispensed medications up to a ceiling per calendar month. During our study GMS patients paid €2.50 per prescription item dispensed, up to a maximum of €25 per month.

Antihypertensive medication are an essential medication that may be particularly susceptible to cost-sharing policies due to the asymptomatic nature of hypertension [2, 8]. Poor adherence to antihypertensive medication is a major contributor to the failure to achieve blood pressure targets [9]. A meta-analysis identified that good adherence is associated with a 19% reduced risk of cardiovascular disease and a 29% reduction in risk of all-cause mortality [10]. Analysis of the Irish national primary-care reimbursement database, indicated that the introduction of the co-payment in 2010 was associated with a 4.8% decrease in antihypertensive adherence with similar decreases observed when the co-payment was further increased in January 2013 [11, 12]. However, there is socioeconomic variability within the GMS population, as eligibility varies with significantly higher income thresholds applying for older patients (Table 1) [13]. People on lower income are considered more susceptible to the effects of co-payments [14, 15]. Thus, it is possible the observed reduction in adherence may have been underestimated for those on lower income. As a result, these patients may be at greater risk of cardiovascular disease and events such as stroke and myocardial infarction due to lower antihypertensive medication adherence [16]. This further compounds existing health inequalities of higher rates of cardiovascular disease and events in socioeconomically deprived groups [17, 18].

Our objective was to 1) evaluate the medication-related financial burden imposed by out of pocket expenses resulting from the GMS co-payment, and 2) to assess the effect of this financial burden on adherence at 12 months, in a cohort of older (> 65 years) hypertensive community dwelling adults.

## Methods

### Study setting, participants and design

We conducted a prospective cohort study, recruiting participants from 106 community pharmacies across the Republic of Ireland between March and May 2014. Pharmacies were selected on the basis of participating in the National Pharmacy Internship Programme. Participants completed a structured telephone interview conducted by trained pharmacy interns and were re-contacted at 12 months to complete a follow-up structured telephone interview. Interviews were subsequently linked to each patients’ pharmacy records. The financial burden of medication costs was evaluated at baseline interview, and antihypertensive medication adherence was assessed at follow-up via a self-report questionnaire and by calculating the proportion of days covered (PDC) from linked dispensing records. Ethical approval for this study was granted by the Research Ethics Committee of the Royal College of Surgeons in Ireland.

### Inclusion and exclusion criteria

Each pharmacy aimed to recruit 15 participants, inviting consecutive patients presenting a prescription for at least one medication for hypertension, aged 65 years or older, community dwelling, able to speak and understand English with no evidence of cognitive impairment as judged by the pharmacist. For this analysis we excluded non-GMS patients.

### Exposure

We assessed medication-related financial burden using a single item from the Adherence Estimator [19], “*I feel financially burdened by out of pocket expenses incurred by medication costs”*, with an accompanying 6-level Likert-type response ranging from *Agree Completely* to *Disagree Completely*. The Adherence Estimator is a three-item questionnaire that evaluates three proximal predictors of adherence, perceived need for medication, perceived concerns about medication and perceived medication affordability. The Adherence Estimator has been psychometrically validated and demonstrated predictive validity with PDC [19, 20]. We focussed on the item, perceived medication affordability, which assigns a score of 2 to *Agree Completely* and *Agree Mostly* and 0 to all other responses. We modified the responses to a 5-level Likert-scale, collapsing the responses *Agree Somewhat* and *Disagree Somewhat* to a single response *Uncertain.* Based on the original scoring method we derived a binary exposure variable, perceived financial burden, combining the two levels that indicated agreement with the statement versus the remaining three levels, which do not indicate agreement. Participants were asked to respond in relation to their antihypertensive medication.

### Outcome

We evaluated adherence to antihypertensive medication at 12-month follow-up using the 8-item Morisky Medication Adherence Scale (MMAS-8). The MMAS-8 is an 8-item measure with 7 yes/no items (e.g. “*Do you sometimes forget to take your medication”*) and one 5-point response scale (“*How often do you have difficulty remembering to take all your medication*”) (© 2007 Donald E. Morisky). Participants responded to these questions in relation to their antihypertensive medication. Higher scores on the MMAS-8 indicate better adherence. The MMAS-8 has been shown to be reliable in estimating adherence to antihypertensive medications and to have predictive validity through associations with blood pressure control [21, 22].

We also evaluated refill adherence as PDC from linked dispensing records [23]. Refill adherence has demonstrated predictive validity in hypertension through significant associations with blood pressure control [24–26]. We calculated PDC by dividing the number of days’ covered by the antihypertensive medication from the date of the first prescription during the observation period to the end of the observation period. Oversupplies at the end of the observation period were excluded. For patients receiving multiple antihypertensive medication, an overall PDC was obtained by averaging PDCs across each class of antihypertensive. PDCs exceeding 1, which indicate oversupplies of medication, were recoded to 1. PDC was rescaled to range from 0 to 100 to aide interpretation of regression results.

### Confounders

Other factors which may also influence adherence including demographics (i.e. age, gender, education), private health insurance, beliefs about medicines, health behaviours (smoking), comorbidities, and medication history were recorded at baseline interview [3]. Age was dichotomised as 65–69 years and 70+ years to reflect age categories for the GMS eligibility income thresholds. In addition to public insurance all Irish patients can purchase private health insurance, which is used to pay for private care in hospitals or from private health professional practices [27], and is a general indicator of higher socioeconomic status. The concerns and necessity-beliefs subscales of the Beliefs about Medicines Questionnaire (BMQ-Specific) were completed at baseline. According to the Necessity-Concerns Framework (NCF), patients are more likely to take their medication if they have stronger medication necessity beliefs and fewer medication concerns [28]. Higher scores on the BMQ-Concerns indicate greater concerns regarding antihypertensive medication; higher scores on the BMQ-Necessity indicate stronger beliefs in the necessity of antihypertensive medication. Multimorbidity was measured as a count of self-reported comorbid conditions, in addition to hypertension [29]. History of heart attack, angina and stroke, were considered important covariates, as adherence to treatment of hypertension for secondary prevention may be higher in these groups [30]. The patient’s medication regimen complexity, which may also influence adherence [31–33], was assessed via a number of variables which were determined at baseline from dispensing records, including the use of multi-dose units packaging (MDUs), the number of regular medicines, the class and number of specific antihypertensive medication, and the dosing frequency. The number of regular medications will also determine the monthly cost of medication and influence perceived medication-related financial burden, as GMS patients paid €2.50 per each item dispensed to a maximum of €25 per month. Additionally marital status was included as the monthly payment ceiling is applied per couple.

### Statistical analyses

Descriptive statistics for the study sample are presented according to medication-related financial burden, and associations evaluated using Pearson’s χ^2^ for categorical and binary variables, and *t*-tests for continuous variables. Separate multivariate linear regression models were used to evaluate the association between medication-related financial burden and adherence (MMAS-8 and PDC), adjusting for confounders described previously. Standard errors were adjusted in regression models using the Sandwich-estimator, due to potential for dependency of observations at the pharmacy-level. Statistical modelling was performed using Stata version 14 (StataCorp College Station, Texas, USA).

## Results

### Participants

**Fig. 1:**
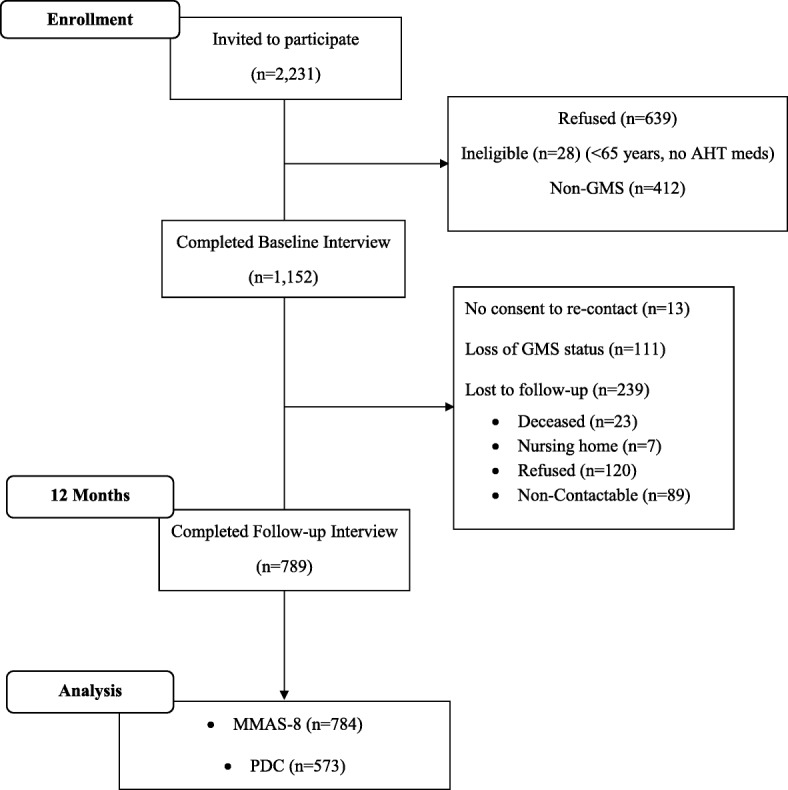
Flow diagram detailing number of patients recruited, numbers excluded from current study, and total number included in current analysis

At baseline, 1564 patients agreed to participate and completed the baseline structured telephone interview. Applying the exclusion criteria, approximately half of participants (*n* = 145) under 70 years and 81% (*n* = 1007) of those 70 years and older were enrolled in the GMS, leaving a final sample of 1152 GMS participants (Fig. 1). The mean age of this sample was 77.4 (*SD 6.0)* years, 44.5% were men and on average participants were taking antihypertensive medication for 11.3 (*SD 9.2*) years.

### Medication-related financial burden

Almost one third of GMS participants, 30.1% (*n* = 345), reported that they completely or mostly agreed that they were financially burdened by out of pocket medication costs. Table 2 outlines associations between baseline participant characteristics and medication-related financial burden. Participants that reported perceived medication-related financial burden were more likely to have lower education attainment, to lack private health insurance, to have had cardio- or cerebrovascular disease (angina, stroke), to have a higher number of co-morbidities, to use more medication, to have a higher daily dose frequency of antihypertensive medication, and to have higher concerns about their antihypertensive medication.

**Table 2:** Summary of sample characteristics by medication financial burden (*n* = 1152)

Socio-Demographics	Financial Burden Yes (*n* = 345)	Financial Burden No (*n* = 803)	*p*
*Age* < 70 years, % (n)	86.6 (46)	87.9 (97)	
70+ years, % (n)	13.4 (298)	12.1 (705)	0.549
Male, % (n)	46.1 (159)	44.1 (354)	0.532
*Education attainment*			
Primary, % (n)	46.3 (156)	31.0 (234)	
Secondary, % (n)	37.4 (126)	45.6 (345)	
Third-level, % (n)	16.3 (55)	23.4 (177)	< 0.001
*Marital Status*			
Married/Partner, % (n)	56.8 (193)	60.0 (467)	
Single/Divorced/Widowed, % (n)	42.6 (147)	38.9 (312)	0.319
Private Health Insurance: % (n)	31.4 (105)	47.8 (371)	< 0.001
Medical History			
Current Smoker, % (n)	10.5 (36)	7.5 (60)	0.098
Heart Attack, % (n)	16.3 (56)	13.5 (108)	0.210
Angina, % (n)	18.9 (65)	12.1 (97)	0.002
Stroke, % (n)	5.8 (20)	2.4 (19)	0.003
No. of comorbidities, mean (*SD*)	2.87 (*1.8*)	2.29 (*1.5*)	< 0.001
Medication History			
No. of regular medicines, mean (*SD*)	7.1 (*3.9*)	6.1 (*3.7*)	< 0.001
Medication repackaged in MDU, % (n)	13.3 (46)	12.1 (97)	0.555
Years on AHT medication, mean (*SD)*	11.2 (*8.7)*	11.4 (*9.4)*	0.795
AHT Dosing Frequency, mean (*SD*)	1.16 (*0.39)*	1.08 (*0.26*)	0.001
AHT Defined Daily Dose, mean (*SD*)	2.62 (*1.96*)	2.60 (2.09)	0.882
Angiotensin acting agents, % (n)	75.1 (256)	75.4 (596)	0.895
Alpha-blockers, % (n)	6.5 (22)	5.6 (44)	0.561
Beta-blockers, % (n)	52.5 (179)	47.1 (372)	0.095
Calcium Channel Blockers, % (n)	45.5 (155)	42.8 (338)	0.406
Diuretics, % (n)	26.7 (91)	32.2 (254)	0.067
BMQ-Specific Concerns, mean (*SD)*	2.28 (*0.63)*	2.17 (*0.59)*	0.004
BMQ-Specific Necessity, mean (*SD)*	3.72 (*0.63)*	3.67 (*0.70)*	0.226

*AHT* = antihypertensive, *BMQ* = beliefs about medication questionnaire, *MDU* = multi-dose units. *n* may be smaller due to missing data across variables (*n*): financial burden (4), age (2), education (55), marital status (29), private health insurance (37), smoking history (8), medical history (1), length of time on AHT medication (137), medication history (17), BMQ-Specific concerns (40), BMQ-Specific necessity (35)

### Adherence

At 12-months, participants were re-contacted and 789 GMS patients (68%) agreed to the follow-up interview. Complete MMAS-8 scores were available for 784 participants and mean self-reported adherence was 7.2 (*SD 1.1).* Using the defined MMAS-8 cut-offs [34], 51.5% of participants reported high adherence (score = 8), 36.2% reported medium adherence (score = 6 < 8) and 12.2% reported low adherence (score < 6). PDC was calculated from linked dispensing records for 573 participants. Dispensing records were missing for 155 participants and a further 61 participants who reported attending other pharmacies were excluded, as medication dispensed elsewhere was not captured. Mean refill antihypertensive adherence for the 12-month follow-up period was 0.94 (*SD 0.11*), with 9.1% (*n* = 52) categorised as non-adherent using the 0.8 (80%) threshold [23, 35].

### Association between financial-burden and adherence

Table 3 details the estimates from separate linear regression models for the association between medication-related financial burden and medication adherence, adjusting for covariates including socio-demographics, multimorbidity, medication use, and medication beliefs. GMS participants with medication-related financial burden at baseline had statistically significant lower self-reported adherence (*β* = − 0.29, 95% CI -0.48 to − 0.11), corresponding to an adjusted mean difference of 3.6%. Similarly financially-burdened participants had lower PDC (*β* = − 2.76, 95% CI -5.65 to 0.14) at 12 months follow-up, however this was not statistically significant.

**Table 3:** Separate multivariate linear regression models estimating the association between medication-related financial burden and self-reported and medication-refill adherence, adjusting for covariates

	Model 1 - MMAS-8			Model 2 - PDC		
	*β*	95% CI	*p*	*β*	95% CI	*p*
Financial Burden	−0.29	−0.48 - -0.11	0.002	−2.76	−5.65 - 0.14	0.062
Age	− 0.15	− 0.46 - 0.16	0.341	1.28	−1.74 - 4.31	0.401
Male	− 0.19	− 0.36 - -0.03	0.019	− 0.68	−3.14 - 1.78	0.585
*Education*						
Secondary	−0.06	− 0.23 - 0.12	0.513	−1.02	−3.70 - 1.65	0.449
Third-Level	0.02	−0.22 - 0.26	0.872	−2.94	−7.38 - 1.50	0.191
*Marital Status*						
Single/Divorced/Widow	−0.07	− 0.25 - 0.11	0.421	0.17	−1.86 - 2.20	0.866
Private Health Insurance	0.01	−0.15 - 0.17	0.896	1.42	−0.79 - 3.64	0.205
Current Smoker	−0.25	−0.56 - 0.07	0.123	−1.84	−6.48 - 2.80	0.432
Heart Attack	−0.18	−0.44 - 0.08	0.169	1.06	−1.99 - 4.11	0.492
Angina	0.09	−0.19 - 0.37	0.532	0.06	−2.12 - 2.24	0.958
Stroke	0.15	−0.33 - 0.64	0.530	−0.89	−7.50 - 5.71	0.789
No. of comorbidities	−0.05	−0.12 - 0.01	0.123	0.07	−0.57 - 0.72	0.829
No. of regular medicines	0.04	0.01–0.07	0.007	0.53	0.17–0.89	0.005
Use of MDUs	−0.32	−0.63 - -0.01	0.041	−0.14	−2.97 - 2.68	0.920
AHT Dosing Frequency	0.07	−0.09 - 0.23	0.397	−0.68	−3.20 - 1.84	0.594
AHT WHO-DDD	0.02	−0.03 - 0.06	0.451	0.01	−0.45 - 0.47	0.975
Angiotensin acting agents	−0.01	−0.24 - 0.22	0.955	−0.45	−3.46 - 2.55	0.764
Alpha-blockers	−0.17	−0.60 - 0.25	0.418	−0.40	−4.04 - 3.24	0.828
Beta-blockers	−0.07	−0.26 - 0.12	0.451	1.13	−0.97 - 3.23	0.287
Calcium Channel Blockers	−0.01	−0.18 - 0.15	0.868	0.52	−1.80 - 2.84	0.657
Diuretics	0.01	−0.16 - 0.17	0.945	−0.98	−3.25 - 1.30	0.396
BMQ-Specific Concerns	−0.12	−0.25 - 0.005	0.059	−0.72	−2.38 - 0.93	0.388
BMQ-Specific Necessity	0.14	0.021–0.27	0.022	−0.05	−1.38 - 1.27	0.936

*AHT* = antihypertensive, *BMQ* = beliefs about medication questionnaire, *MDU* = multi-dose units. Standard errors were adjusted using the Sandwich-Estimator due to potential clustering effect at the pharmacy level. Model 1 (*n* = 653) and Model 2 (*n* = 481); n is smaller due to missing data across covariates

Permission to use the MMAS scales is required. Reproduction and distribution of the MMAS is protected by US copyright laws. A license agreement to use the scale is available from: Donald E. Morisky, ScD, ScM, MSPH, Professor, Department of Community Health Sciences, UCLA School of Public Health, 650 Charles E. Young Drive South, Los Angeles, CA 90095–1772, dmorisky@gmail.com.

### Participant attrition

For the MMAS-8 analysis, participants lost to follow-up were more likely to have higher education attainment (*χ*^2^(2) = 16.0, *p* < 0.001), however attrition was not related to medication-related financial burden nor baseline MMAS-8 scores. For the PDC analysis, the majority of missing dispensing records at follow-up were related to study logistics, resulting from the absence of a pharmacy intern at the pharmacy of recruitment to link dispensing records at 12-month follow-up (*χ*^2^(1) = 70.6, *p* < 0.001). Compared to participants included in the PDC analysis, those excluded were less likely to report medication-related financial burden (*χ*^2^(1) = 3.9, *p* = 0.047), to have higher education attainment (*χ*^2^(2) = 20.8, *p* < 0.001), to report fewer co-morbidities (*t*(1149) = − 3.09, *p* = 0.002), to use fewer regular medication (*t*(1133) = − 6.47, *p* < 0.001), and to have lower antihypertensive refill-adherence at baseline (*t*(1005) = − 5.10, *p* < 0.001).

## Discussion

### Principal findings

In this prospective cohort study, almost one third of publically insured older community dwelling antihypertensive users perceived that they were financially burdened by the GMS medication co-payment policy. Medication-related financial burden had a subsequent negative impact on self-reported antihypertensive adherence at 12 months, although the decrease in refill adherence at 12 months was not statistically significant.

### Findings in the context of previous literature

The cost of medication has been long-established to be a financial barrier to adherence [3, 4], with systematic reviews identifying direct prescription cost-sharing and co-payment policies to have a negative effect on adherence to medication [2, 6]. The introduction of the co-payment to the GMS scheme in Ireland led to a 5% decrease in antihypertensive adherence, and when the co-payment was further increased, subsequent decreases in adherence, albeit of smaller magnitude, were also observed [11, 12]. This finding was however limited due to the potential socioeconomic variability within the GMS population resulting from varying eligibility income thresholds. As a result of the variability, the negative impact to adherence for those on lower incomes may have been underestimated [14]. We found a third of older antihypertensive users perceive to be financially burdened by the cost of the GMS co-payment. Medication-related financial burden was also associated with a number of indicators of lower socio-economic status including lower education attainment, lower proportion of private health insurance, higher levels of co-morbidity including chronic diseases such as angina and stroke. Those who reported medication-related financial burden at baseline subsequently reported 3.6% lower adherence at 12 months. This is an indicator that for those on lower income, the co-payment has had a greater impact on adherence than the previously published estimates [11, 12]. Similarly, refill adherence was 2.8% lower in participants reporting financial burden, however this was not statistically significant.

Poorer adherence to antihypertensive medication is associated with a higher risk of adverse health outcomes, such as stroke [10, 36–38]. It has been estimated that improving adherence by 15% may reduce the hazard of stroke by 8–9% [38]. In addition to known health inequalities [17, 18], patients financially-burdened by the co-payment may thus be at further increased risk of stroke due to lower antihypertensive adherence. Budgetary savings achieved by these co-payments may ultimately be offset by longer-term healthcare costs [16, 39, 40]. For example long-term average cost of care following stroke is estimated between €4300 - €35,600, depending on stroke severity [41]. Alternative policy approaches to patient co-payments should be considered by the Irish government, such as those based on ability to pay and disease severity [42]. Prior to the introduction of the current co-payment system, the Economic and Social Research Institute (ESRI) in Ireland, suggested a graduated co-payment system based on ability to pay (e.g. income) and severity of disease (e.g. primary or secondary prevention of CVD) [42]. Alternatively, a value-based healthcare approach could be considered, whereby patients are not required to contribute a co-payment towards treatments of known cost-effectiveness, yet a reduction in third-party payer costs is maintained for non-essential medication [1, 43].

### Strengths and limitations

There are number of strengths to this study including the recruitment of community-dwelling older people from a nationally representative sample of pharmacies in Ireland, although this non-probabilistic sampling method may have introduced selection bias. However, consecutive sampling provides structure and additional rigour, ensuring all potential participants can be enrolled, and will produce a more representative sample of the target population than convenience sampling. For the MMAS-8 analysis, participants with higher education attainment were more likely to be lost to follow-up, however attrition was largely related to the study logistics, whereby an intern was not present to facilitate follow-up in a number of pharmacies. Nevertheless, attrition was not related to baseline self-report adherence or perceived financial burden and is unlikely to have biased estimates for this analysis. However for the PDC analysis there was differential attrition, with participants missing PDC measurements at follow-up less likely to report financial burden, and to have lower PDC at baseline which may have biased associations.

Further strengths include the prospective evaluation between exposure and outcomes, with exposure measured prior to outcomes. However, there are some limitations to measurement of these variables. Self-reported adherence may be subject to recall bias and social desirability bias resulting in overestimation of adherence [44]. Similarly, as we have previously reported PDC may overestimate adherence in this publically insured population due to the complexity of reimbursement processes, and the presence of workflow procedures to manage MDU repackaging [45, 46]. Although lower PDC was observed at 12 months in the financially burdened group, the non-significant statistical finding may result as an attenuation of the effect size due to over-estimation of refill adherence, differential attrition and the smaller sample size for this analysis. Furthermore, the PDC variable was highly left-skewed, resulting from overestimation of refill-adherence, and post-regression diagnostics revealed violation of the assumption of the normality of residuals. Additional limitations include the use of a single item to assess medication-related financial burden. However, this item was drawn from a psychometrically validated adherence questionnaire, which includes two further items regarding perceived medication necessity and concerns [19, 20]. Although an altered response scale was employed, the scoring method for this item was not altered, which results in a binary variable. Dichotomisation of variables, however, may result in loss of statistical information, reducing study power. A sensitivity analysis treating medication-related financial burden as a continuous variable based on the original Likert-type responses, obtained similar results (Additional file 1). However, caution must be used when treating Likert-type responses as continuous variables, as the distance between each step on the Likert response scale may not infer the same meaning to the respondent. Finally, residual confounding may remain although a number of theoretical factors, which may also influence both medication-related financial burden and adherence were controlled for, including medication beliefs using the BMQ-Specific, education attainment and private health insurance as a proxy indicator of socioeconomic status, marital status and the number of regular monthly medication which affect the monthly-ceiling for out of pocket costs for medication.

The generalisability of these findings may also be limited; this cohort consisted of hypertensive adults 65 years and over – the association between medication-related financial burden and adherence may differ for younger GMS patients but also for other long-term illnesses. However this should not diminish the importance of these findings; hypertension is a highly prevalent condition which increases with age [47] and is a leading cause of cardiovascular disease, the largest cause of morbidity and mortality globally [48, 49]. In Ireland cardiovascular disease is the most common cause of mortality [50], with approximately two-thirds of adults over 50 years developing hypertension and fewer than a third achieving optimal blood pressure control [51]. Lower antihypertensive adherence is associated with poorer cardiovascular outcomes for patients and longer-term healthcare costs [16]. However, further research confirming these findings using an objective method to evaluate adherence and examining medication-related financial burden for other medication classes (e.g. statins) is warranted.

## Conclusion

In conclusion we found that a co-payment of €2.50 represents a financial barrier to antihypertensive adherence for a third of a publically insured population. The introduction of the GMS co-payment policy is likely to have had a larger impact on medication adherence for GMS patients financially burdened by the co-payment cost. Reductions in adherence to essential medications such as antihypertensive medication can lead to poorer health outcomes and short-term savings achieved from the co-payment may potentially be offset by longer-term higher healthcare costs [16]. Since 2018 the co-payment has been reduced to €2.00, however alternative policies which exempt medication such as antihypertensive from this charge should be considered [43]. Further research on the impact of the co-payment policy has had on patient outcomes and long-term healthcare costs is warranted.

## Additional file

Additional file 1: Sensitivity analysis using continuous exposure variable. (DOCX 19 kb)

## Abbreviations

AHT: Antihypertensive medication
BMQ: Beliefs about Medication Questionnaire
GMS: General Medical Scheme
MDU: Multidose unit
MMAS-8: 8-item Morisky Medication Adherence Scale
PDC: Proportion of Days Covered
