# Supplementary material for: Impact of financial burden, resulting from prescription co-payments, on antihypertensive medication adherence in an older publically insured population
Source: BMC Public Health. 2018 Nov 20;18:1282. doi: 10.1186/s12889-018-6209-8 (PMC6247632; doi:10.1186/s12889-018-6209-8)
Supplement: Supplementary file 1 — Sensitivity analysis using continuous exposure variable. (DOCX 19 kb) [file 12889_2018_6209_MOESM1_ESM.docx]

# **Additional file 1 Sensitivity analysis using continuous exposure variable**

In the primary analysis mediation-related financial burden was treated as a binary variable, based on the scoring algorithm derived from the psychometric testing underpinning this questionnaire item (Adherence Estimator). However, dichotomisation results in loss of important statistical information and can lead to both type I and type II errors. Thus, a sensitivity analysis treating the original responses to the question as a continuous variable was undertaken (1=strongly disagree, 2=disagree, 3=uncertain, 4=agree, 5=strongly agree). Separate multivariate linear regression models were used to evaluate the association between medication-related financial burden and adherence (MMAS-8 and PDC), adjusting for covariates listed in table 1. Standard errors were adjusted in regression models using the Sandwich-estimator, due to potential for dependency of observations at the pharmacy-level. Similar results for the influence of medication-related financial burden on adherence to the primary analysis were observed. Quantitatively the effect sizes are similar, while there is reduced error in the estimates for the PDC analysis. However caution must be used when treating Likert scale responses as continuous variables as it cannot be assumed that distances between responses on a Likert scale are identical (i.e. does the respondent give the same level of meaning from moving from strongly agree to agree, as moving from agree to uncertain). Treating the Likert-scale as a multilevel categorical variable was also considered, however this would further reduce statistical power.

**Table S1. Sensitivity analysis using continuous medication-related financial burden variable. Separate multivariate linear regression models estimating the association between medication-related financial burden and self-reported and medication-refill adherence, adjusting for covariates.**

|  | **Model 1 - MMAS-8** | | | **Model 2 - PDC** | | |
| --- | --- | --- | --- | --- | --- | --- |
|  | ***β*** | **95% CI** | ***p*** | ***β*** | **95% CI** | ***p*** |
| Financial Burden | -0.09 | -0.16 - -0.02 | 0.012 | -1.03 | -2.03 - -0.03 | 0.044 |
| Age | -0.14 | -0.45 - 0.17 | 0.366 | 1.39 | -1.65 - 4.43 | 0.364 |
| Male | -0.20 | -0.36 - -0.03 | 0.019 | -0.72 | -3.18 - 1.74 | 0.564 |
| *Education*  Secondary  Third-Level | -0.05  0.02 | -0.22 - 0.12  -0.22 - 0.26 | 0.556  0.885 | -1.08  -3.11 | -3.76 - 1.61  -7.58 - 1.35 | 0.428  0.169 |
| *Marital Status*  Single/Divorced/Widow | -0.07 | -0.25 - 0.11 | 0.885 | 0.23 | -1.82 - 2.28 | 0.822 |
| Private Health Insurance | 0.01 | -0.15 - 0.17 | 0.454 | 1.37 | -0.87 - 3.60 | 0.228 |
| Current Smoker | -0.24 | -0.56 - 0.08 | 0.895 | -1.92 | -6.65 - 2.81 | 0.422 |
| Heart Attack | -0.18 | -0.43 - 0.08 | 0.133 | 1.08 | -1.99 - 4.14 | 0.487 |
| Angina | 0.08 | -0.20 - 0.36 | 0.170 | -0.03 | -2.23 - 2.16 | 0.976 |
| Stroke | 0.15 | -0.34 - 0.63 | 0.557 | -0.90 | -7.52 - 5.73 | 0.789 |
| No. of comorbidities | -0.05 | -0.12 - 0.01 | 0.551 | 0.08 | -0.58 - 0.73 | 0.818 |
| No. of regular medicines | 0.04 | 0.01 - 0.07 | 0.109 | 0.53 | 0.17 - 0.89 | 0.005 |
| Use of MDUs | -0.32 | -0.63 - -0.02 | 0.007 | -0.22 | -3.03 - 2.59 | 0.876 |
| AHT Dosing Frequency | 0.07 | -0.1 - 0.23 | 0.037 | -0.69 | -3.20 - 1.82 | 0.587 |
| AHT WHO-DDD | 0.02 | -0.02 - 0.06 | 0.426 | 0.01 | -0.44 - 0.47 | 0.957 |
| Angiotensin acting agents | 0.00 | -0.23 - 0.23 | 0.428 | -0.46 | -3.46 - 2.55 | 0.763 |
| Alpha-blockers | -0.17 | -0.59 - 0.26 | 0.987 | -0.46 | -4.06 - 3.13 | 0.798 |
| Beta-blockers | -0.07 | -0.26 - 0.12 | 0.433 | 1.06 | -1.02 - 3.14 | 0.313 |
| Calcium Channel Blockers | -0.02 | -0.19 - 0.15 | 0.450 | 0.48 | -1.85 - 2.81 | 0.683 |
| Diuretics | 0.01 | -0.16 - 0.17 | 0.802 | -0.93 | -3.21 - 1.34 | 0.417 |
| BMQ-Specific Concerns | -0.11 | -0.24 - 0.01 | 0.922 | -0.60 | -2.29 - 1.10 | 0.484 |
| BMQ-Specific Necessity | 0.14 | 0.02 - 0.27 | 0.078 | -0.06 | -1.39 - 1.27 | 0.929 |

AHT=antihypertensive, BMQ=beliefs about medication questionnaire, MDU=multi-dose units. Standard errors were adjusted using the Sandwich-Estimator due to potential clustering effect at the pharmacy level. Model 1 (n=653) and Model 2 (n=481); n is smaller due to missing data across covariates.
